# Diffusion of hydrophobin proteins in solution and interactions with a graphite surface

**DOI:** 10.1186/2046-1682-4-9

**Published:** 2011-04-21

**Authors:** Paolo Mereghetti, Rebecca C Wade

**Affiliations:** 1 Heidelberg Institute for Theoretical Studies (HITS) gGmbH, Schloß-Wolfsbrunnenweg 35, 69118 Heidelberg, Germany; 2 Interdisciplinary Center for Scientific Computing (IWR), University of Heidelberg, Im Neuenheimer Feld 368, D-69120 Heidelberg, Germany

## Abstract

**Background:**

Hydrophobins are small proteins produced by filamentous fungi that have a variety of biological functions including coating of spores and surface adhesion. To accomplish these functions, they rely on unique interface-binding properties. Using atomic-detail implicit solvent rigid-body Brownian dynamics simulations, we studied the diffusion of HFBI, a class II hydrophobin from *Trichoderma reesei*, in aqueous solution in the presence and absence of a graphite surface.

**Results:**

In the simulations, HFBI exists in solution as a mixture of monomers in equilibrium with different types of oligomers. The oligomerization state depends on the conformation of HFBI. When a Highly Ordered Pyrolytic Graphite (HOPG) layer is present in the simulated system, HFBI tends to interact with the HOPG layer through a hydrophobic patch on the protein.

**Conclusions:**

From the simulations of HFBI solutions, we identify a tetrameric encounter complex stabilized by non-polar interactions between the aliphatic residues in the hydrophobic patch on HFBI. After the formation of the encounter complex, a local structural rearrangement at the protein interfaces is required to obtain the tetrameric arrangement seen in HFBI crystals. Simulations performed with the graphite surface show that, due to a combination of a geometric hindrance and the interaction of the aliphatic sidechains with the graphite layer, HFBI proteins tend to accumulate close to the hydrophobic surface.

## Background

Hydrophobins are small (7-15 kDa) proteins produced by filamentous fungi [1]. They perform a range of biological roles including coating of spores and surface adhesion [2,3]. Except for Botrytis cinerea, where their function is unknown [4], hydrophobins lower the surface tension of water so that fungal hyphae can penetrate the air-water interface and grow outside aqueous media [5]. To carry out these functions, they rely on unique surface/interface binding properties [1,3,6-8]. Besides their peculiar surface properties, which make them the most powerful surface-active proteins known [3], they also display unusual behaviour in solution as they form different kinds of oligomers, depending on the conditions and on the hydrophobin type [9,10]. Hydrophobins have been divided into two classes, class I and class II, based on the hydropathy profile of the amino-acid sequence [1]. This classification is also consistent with other properties. In particular, class I hydrophobins are more resistant to dissociation using solvents and detergents than class II hydrophobins. Furthermore, class I hydrophobins tend to form rodlet-like aggregates at interfaces, whereas class II hydrophobins do not. Although, the different types of hydrophobins show a great variability in aminoacid sequence (with sequence identity sometimes as low as 30% [7]), they all present a characteristic pattern of four disulfide bridges formed by eight conserved cysteines [11]. This disulfide bridge pattern is common to all known class I and class II hydrophobins. For a recent review on hydrophobins, see ref. [3].

The hydrophobin HFBI from *Trichoderma reesei*, which will be considered in this study, belongs to class II hydrophobins and its three-dimensional structure has been determined by X-ray crystallography by Hakanpää et al. at 2.1 Å resolution [12]. HFBI has an amphipathic structure, with a large (≈750 Å^2^) solvent-exposed hydrophobic patch containing aliphatic residues and a hydrophilic region composed of polar residues. It is likely that the unfavorable exposure of many hydrophobic residues to the aqueous solvent, is compensated by the four disulfide bridges [3,9].

In the crystal structure, HFBI forms homotetramers. In solutions, HFBI forms oligomers in a concentration-dependent manner, and in particular, it has been shown that dimers and tetramers are present in aqueous solution at protein concentrations (2-20 g/L) [13]. At protein concentrations below 2 g/L, HFBI is monomeric in solution [13,14].

Hydrophobins adsorb on to various types of surfaces, forming regular structures (e.g. hexagonal patterns) or randomly aligned rodlets [3,8,10,15-17]. In a growing number of works, hydrophobins are applied for surface modification and in biosensor development [17-20]. The surface and interfacial activity of HFBI proteins has been studied from experimental [8,13,16] and theoretical [21,22] points of view. From these studies, a general model for how hydrophobins function has emerged. Hydrophobins are soluble in aqueous solution and they form different types of oligomers. Close to an interface, the oligomers dissociate and adsorb on to the surface. The secondary structure of HFBI does not change upon adsorption or self-assembly [8,23]. However minor changes in the orientations of sidechains [8] or loops [12] do occur. It has been suggested that these local rearrangements and, in particular, the conformational change of loop 60-66, are induced by multimer formation [12].

The aim of this work is to shed light on the mechanisms of self-association of HFBI in solution and its adsorption onto a hydrophobic surface. A detailed understanding of these mechanisms can be relevant not only for a better understanding of the biological function of hydrophobins but also for the potential biotechnological application of these macromolecules.

In the next section, we discuss the results obtained from simulations of HFBI solutions at different concentrations. Then, results on the interaction of HFBI solutions with a graphite surface are presented.

## Results and Discussion

### Solution properties of HFBI

The properties of HFBI in aqueous solution were studied by means of simulations at protein concentrations of 2, 5, 10 and 20 g/L. According to the experimental conditions used in ref. [14], a pH of 5 and ionic strength of 50 mM were assumed. Two sets of simulations were done at each protein concentration. In one set, all proteins were in conformation A (corresponding to chain A in the crystal structure) while in the second set, a mixture of 50% conformation A and 50% conformation B (corresponding to chain B in the crystal structure) was simulated. The reason for this, as described in the Methods section, is that the four chains in the HBFI tetramer observed in the asymmetric unit of the crystal structure can be divided into two groups based on the conformation of the second *β*-hairpin (loop 60-66) and on their electrostatic potential [12].

#### Oligomerization

**Figure 1:**
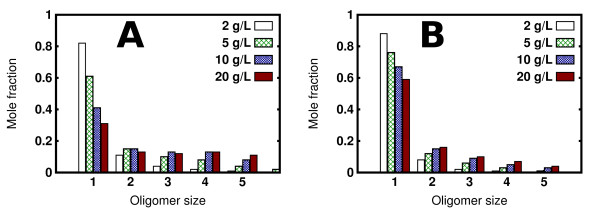
**Oligomerization of HFBI in aqueous solutions**. Fractions of monomeric *χ*_1 _and higher oligomeric states *χ_n _*observed in the simulations. Panel A refers to the simulations performed with 512 A chains while panel B shows the results for mixed chain (256 chain A + 256 chain B) simulations. Four different protein concentrations, indicated on the figure, were tested.

Using the definition of oligomers described in the Methods section, we computed the fractions of HFBI molecules in different oligomeric states (from monomers to pentamers) observed in the simulations (see Figure 1). Considering the simulations performed with conformation A only (Figure 1A), it can be observed that, whereas the fraction of dimers is approximately constant, the fractions of higher order oligomers increases with the protein concentration.

**Figure 2:**
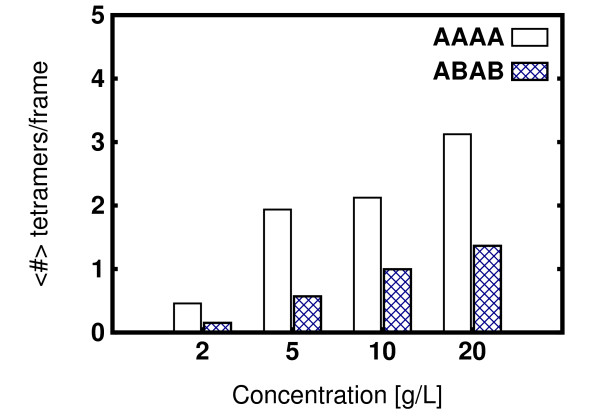
**Tetrameric content**. Average number of tetramers per frame found in mixed conformation (256 chain A + 256 chain B) simulations. Three types of tetramers were distinguished (see Methods): chain A only (AAAA), chain B only (BBBB) and mixed tetramers formed by two chain A and two chain B (ABAB) chains. In the latter case, any permutation was assumed to be identical. The average number of tetramers composed of chain B monomers only was not reported in the figure because it was below 0.1 for all concentrations.

For the mixed chain A and chain B simulations, it can be seen (Figure 1B) that the fractions of oligomers are generally lower than in the chain A only simulations. Moreover, all the oligomer fractions (monomers to pentamers) show a concentration dependence. This suggests that oligomerization depends on a structural rearrangement of HBFI, as was previously proposed [12]. In particular, Hakanpää et al. explained the different conformations found in the crystal structure as resulting from a structural change after HBFI tetramer formation [12]. Analyzing the tetramers formed in the inhomogeneous system, we find that chain B tends to disfavour the formation of tetramers. This is shown in Figure 2 where the fractions of the different types of tetramer formed are shown. The two conformations essentially differ only in the structure of the second *β*-hairpin (loop 60-66), which in chain B is present in a solvent-exposed conformation, as well as in the orientation of some sidechains. As was mention in ref. [12], it would not be possible to form the tetramers observed in the crystal structure if all the monomers were in the chain B conformation because of steric clashes of the extended conformation of loop 60-66. Despite the structural rigidity of HFBI due to the four disulfide bridges, minor structural changes occur, and seem to be important for the oligomerization process. It is not clear whether the process of formation of the tetramers can be cast as an induced fit or a conformational selection model. This question could be addressed by performing all-atom molecular dynamics simulations of the tetrameric encounter complexes generated in our Brownian dynamics simulations.

#### Tetrameric encounter complexes

**Figure 3:**
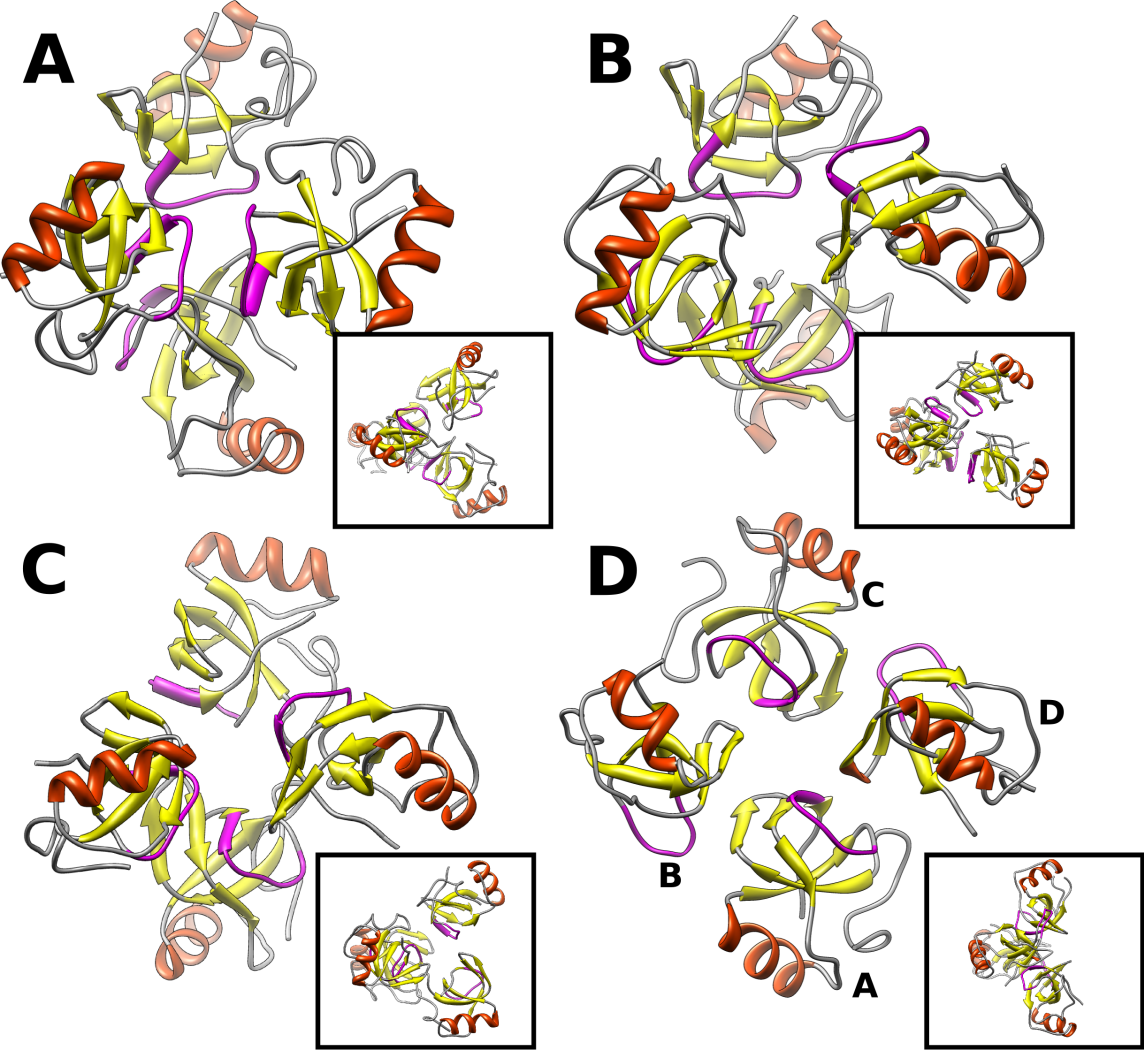
**Shape of the tetramers**. Ribbon representation of the centroids of the first three most populated clusters (A, B, C) of encounter complexes obtained in the simulation at 5 g/L with only conformation A. In D, the ribbon trace of the crystal structure is shown. The insets show the corresponding structure rotated clockwise by 90° about the vertical axis. The loop 60-66, which plays an important role in tetramer formation, is shown in mauve.

**Figure 4:**
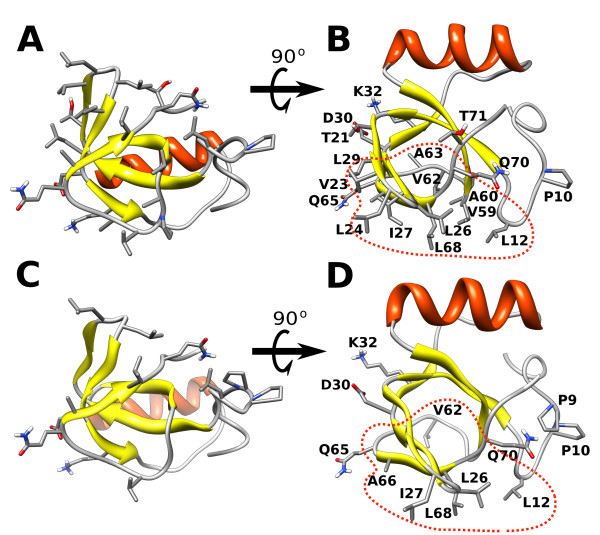
**The most frequently observed atomic contacts in the simulations**. The residues with a contact probability larger than a defined threshold (see Methods Section) are shown for protein-protein (A, B) and protein-surface (C, D) contacts. A red dotted line is drawn around the residues which form the hydrophobic patch.

From the simulations, all tetramers were collected from snapshots at 1 ns time intervals and then clustered to identify the structures of the tetramer that occur with the highest probabilities in the simulations. In Figure 3A, B, C, structures corresponding to the centroids of the first three most populated clusters obtained in the simulation of HFBI chain A at 5 g/L are shown. The clusters show considerable structural variability with backbone RMSD within the cluster of ≈ 12.0 Å. 15 clusters were obtained with more than 30 members and the top three had about 90 each. The crystal structure of HFBI is also shown (Figure 3D). The arrangement of the monomers in the tetramers obtained from the simulation, differs from the crystal structure and shows large variability. However, the interactions of the four monomeric units always occur via the hydrophobic region (outlining red dotted line in Figure 4B, D), and, as in the crystal structure, the two dimers forming the tetramers are perpendicular to each other (see insets in Figure 3).

The tetramers identified in the simulations can be regarded as encounter complexes. The crystal and the solution structures differ due to several reasons. First, in general, the crystal structure may be different from the structure in solution because of the crystallization procedure. A structure of the tetramer of HFBI in solution was derived in the work of Kisko et al. [14] using small angle X-ray scattering. In that work, the authors obtained a low resolution model in which the monomeric units composing the tetramers were arranged in a somewhat different way that the crystal structure; the two pairs of dimers lay almost in the same plane and the resultant modelled tetramer was flat [14]. Cluster analysis of the tetramers revealed that flat tetramers do not occur in our simulations. Another possibility, which explains the differences in the tetramers we found in simulations from the crystal structure, results from analysis of the inter-atomic contacts between the monomeric units as described later.

#### Electrostatic properties

**Figure 5:**
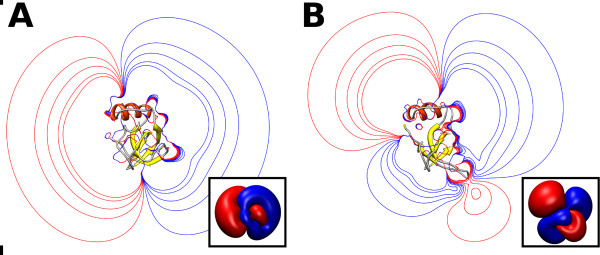
**Electrostatic potentials**. Electrostatic isopotential lines (± [0.01, 0.03, 0.05, 0.08, 0.1] kcal/mol/*e*) for chain A (panel A) and chain B (panel B) of HFBI. Isolines are shown for the cross-section passing through the center of the protein. Three-dimensional isosurfaces at 0.01 kcal/mol/*e *are shown in the insets. The electrostatic potential was computed at pH 5 and 50 mM IS by solving the linearized Poisson Boltzmann equation using UHBD [35] (see Methods Section for details).

**Figure 6:**
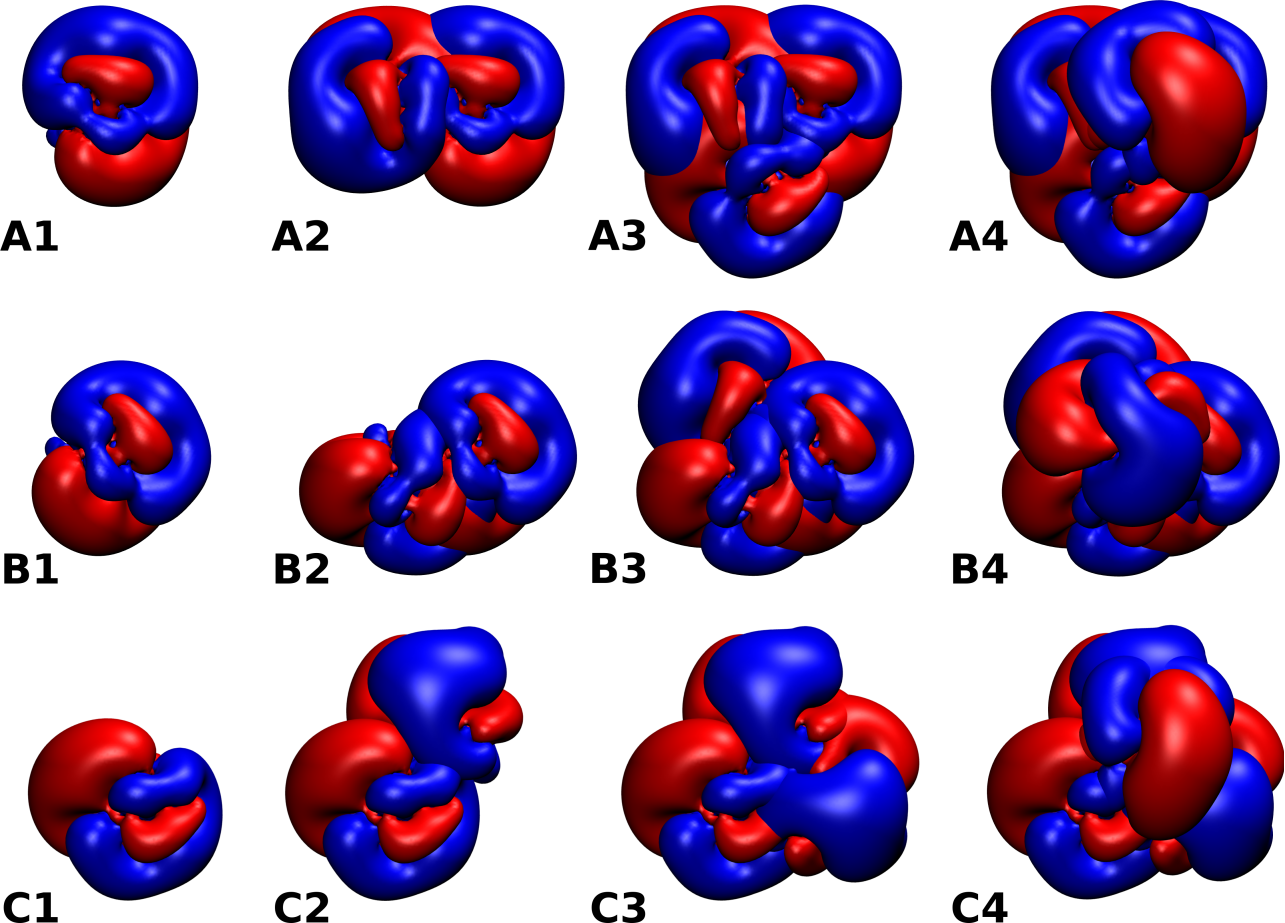
**Electrostatic potential of HFBI encounter complexes**. Three-dimensional isosurfaces are shown at ± 0.05 kcal/mol/*e*. The encounter complexes correspond to those in Figure 3. The columns show the electrostatic potential for 1,2,3 and 4 HFBI monomers for the corresponding homotetrameric encounter complex.

Differences in the configuration of the loop 60-66 and in the sidechain orientations, lead to a predominantly dipolar electrostatic potential for chain A(C) and a quadrupolar electrostatic potential for chain B(D). Comparison of the electrostatic potentials of conformation A and of conformation B of the HFBI is shown in Figure 5. The different electrostatic potential of conformation B is one contribution to the reduced occurrence of tetramers in the simulations with the chain B present (Figure 2). Examination of the electrostatic potential of the identified tetrameric encounter complexes (Figure 6), shows that the magnitude of the electrostatic potential in the central region of the tetramer (corresponding to the hydrophobic patch) is always lower than in the outer region. It is likely that the monomers approach by keeping regions with the same sign electrostatic potential apart. This allows the monomers to orient with their hydrophobic patches facing each other. When the monomeric units are close enough and in the correct orientation, hydrophobic desolvation energies prevail and allow the formation of the encounter complex. In the case of monomers with a quadrupolar electrostatic potential, i.e. chain B, the correct orientation with the four hydrophobic surfaces interacting with each other could not be achieved.

#### Protein-protein contacts

**Figure 7:**
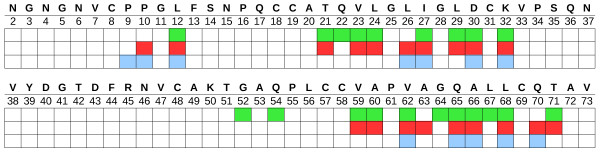
**Interfacial residues**. The sequence of the HFBI chain A is given with interfacial residues shown by colored squares. Interfacial residues in the crystal structure were identified using ePISA [41] and are shown in green. Protein-protein and protein-graphite contact residues are highlighted in red and cyan respectively. As described in the Method Sections, the distance cutoff (*d*_c_) was set to 4.5 Å and 4.0 Å for the definition of the protein-protein and protein-graphite contacts respectively.

A contact analysis was performed to determine contact probabilities for each atom on the protein surface. The residues with a contact probability larger than a defined threshold (see Methods Section) were identified, see Figure 4A, B. Among them, 11 are aliphatic residues located on the hydrophobic patch (red dotted line in Figure 4B, D. Some polar/charged residues (Thr21, Asp30, Lys32, Gln65, Gln70, Thr71) are also present, which are probably involved in lateral interactions between the monomeric units within the tetramer. The amino-acids found correspond almost completely with the interfacial amino-acids found in the crystal structure (see Figure 7). The differences between the prevalent quaternary structures found in the simulations and the arrangement seen in the crystal structure can also be explained by considering the relevance of some of the contacting polar amino-acids for the stabilization of the complex. Firstly, in the crystal structure, a zinc ion coordinates the Asp30 of one chain with the corresponding Asp30 of another chain. Secondly, there is a water bridge between the carboxyl group of the Gln65 of chain C and the amine group of the corresponding Gln65 of chain A. The omission of the explicit modelling of solvent and ions in our simulations may affect polar short range interactions, and thus, prevent the monomers from arranging in the quaternary structure observed in the crystal. Another clearly important factor that should be taken into account is that the formation of the crystallographic arrangement depends on structural relaxation which is not accounted for in the simulations with chain A only.

### Hydrophobic surface interactions

#### Interaction with graphite layer

The interaction with a Highly Ordered Pyrolytic Graphite (HOPG) layer was studied by performing Brownian dynamics simulations of 16 hydrophobin molecules at 20 g/L concentration in the presence of a graphite layer. The HOPG layer was chosen, following Szilvay et al. [13], for its property of being flat and completely hydrophobic. Since the graphite is uncharged and hydrophobic, electrostatic interactions between proteins and the HOPG layer were not modelled; only the non-polar desolvation term and soft-core repulsion contributed to their interaction as described in the Methods Section. The oligomerization properties in simulations with and without the HOPG layer were compared. In the simulations with the surface, the fraction of higher order oligomers was higher than in the simulations without the graphite layer and the oligomers form in a layer very close to the graphite. This phenomenon can be explained by considering the distribution of hydrophobins in the simulation volume.

#### HFBI distribution in the simulation volume

**Figure 8:**
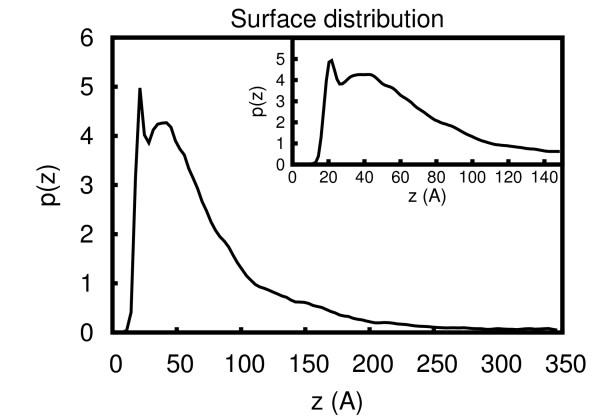
**Distribution of HBFI molecules above the hydrophobic surface**. Distribution of proteins with respect to the distance from the surface in the z direction. The surface of graphite layer is at z = 0.0 Å (see Methods Section for details). The initial portion of the curve (z = [0-150] Å) is shown in the inset.

In the simulations with the graphite layer, the proteins tend to remain close to the surface. The reason is two-fold. First, a geometric hindrance influences the diffusion of the molecules close to the surface because of the removal of a degree of freedom. Second, the favourable contribution of the non-polar desolvation term keeps the proteins near the surface. A plot of the distribution of the center of geometry of the proteins with respect to the surface shows a first sharp peak at 20 Å from the center of the protein to the graphite surface followed by another broader peak around 40 Å from the surface, see Figure 8. Considering a hydrodynamic radius of HFBI of 15 Å, the distance from the protein surface to the graphite surface is 5 Å and 25 Å for the first and the second peaks, respectively. This can be interpreted as showing the proteins arranged in two partially overlapping layers. This is consistent with the model derived by Kisko at al. [8] in which HFBI proteins organize in helical rings of dimers where the two monomers are at a center to center distance of 20 Å from each other. However, in our simulations, the proteins do not arrange in a regular fashion as described in ref. [8]. This can be explained by considering that in Brownian dynamics simulations at constant temperature, the molecules keep fluctuating and cannot form crystallized patterns. The higher surface affinity shown by hydrophobins compared to solution association [16] can be due to the increased local effective concentration close to surfaces arising from steric and hydrophobic effects as explained above.

#### Protein-graphite contacts

Following the same procedure as for the protein-protein interactions, the residues involved in contact with the graphite surface were identified (see Figure 4C, D). As described in the Method Section, the parameter *dc *(distance cutoff) was set to 4.0 Å instead of 4.5 Å in the protein-protein case. Indeed, studies of peptide adsorption to a HOPG layer revealed that the residues can come very close to the surface (≈5 Å from the peptide backbone) [24,25]. The interaction with the graphite surface occurs mainly via the hydrophobic region. The hydrophilic side of the protein tends to remain away from the surface due to the unfavorable desolvation energy of polar/charged residues. It has been found that the adsorption of peptides on HOPG layers is driven by the hydrophobic effect arising from the interaction of methylene groups in amino-acids sidechains with the graphite layer [25].

In the simulations, we identified six aliphatic residues interacting with the graphite; three leucines (Leu12, Leu26, and Leu68), one isoleucine (Ile27) one alanine (Ala66) and one valine (Val62). In an earlier molecular dynamics simulations study, five residues were found to drive the adsorption of HFBII (from *Trichoderma reesei *) on a silicon surface: Leu19, Leu21, Ile22, Ala61 and Leu63 (numbering based on the sequence of HFBII) [22]. Superimposing the structure of HFBI on HFBII, we found that Leu26(21), Ile27(22), Ala66(61) and Leu68(63) are common to the two sets (in parenthesis is the corresponding number in the HFBII sequence). In ref. [22], the authors also found that an aspartic acid (Asp59, HFBII numbering), which is substituted by a glycine in HFBI, determines the orientation of the adsorbed HFBII. In particular, Asp59, which sticks out from the boundary of the hydrophobic patch, drives the tilting of the hydrophobic patch to maximize the desolvation of aliphatic residues and allow solvation of Asp59 [22]. The absence of Asp59 in HFBI leads to a different adsorption orientation. It is probable that the slightly lower binding affinity of HFBII [15] is related to the presence of Asp59 which reduces the contact area between the hydrophobic patch and the hydrophobic surface. From a multiple sequence alignment of class II hydrophobins [6], it is interesting to note that the aminoacid at position 59 (HFBII numbering) is conserved as a glycine with only two exceptions, in HFBII and MAG (from *Magnaporthe grisea *), which have an aspartic acid instead.

In the simulations, some polar and charged aminoacids (Lys32, Asp30, Gln65, Gln70) were found to interact with the graphite layer, however, it is highly probable that these contacts were identified solely due to the extended conformations of their sidechains which stick out from the protein surface.

## Conclusions

In this paper, Brownian dynamics simulations of solutions of the class II hydrophobin, HFBI, from *Trichoderma reesei *were performed. Simulations were carried out at several protein concentrations from 2 g/L to 20 g/L. Moreover, a graphite surface model was included in the simulations and the diffusional association of HFBI proteins to the graphite layer was studied.

In our simulations, hydrodynamic interactions were neglected since in dilute regimes (<0.1 volume fraction) it has been found that for almost spherical unconnected particles, solvent correlations do not affect the dynamic properties significantly. In particular, the experimental concentration dependent diffusion coefficients for different protein solutions can be qualitatively reproduced with Brownian dynamics simulations without hydrodynamic interactions [26-28]. The dynamics of the tetramerization process and the protein-surface adsorption may be affected by hydrodynamic interactions [29,30].

Moreover, as discussed in the main text, the absence of explicit modelled solvent and ions may prevent exact reproduction of the bound state shown in the crystal structure. Despite these limitations, we found from the simulations that HFBI in solution exists as a mixture of different oligomeric states which form in a concentration dependent manner [14]. The formation of stable tetramers, which is the most abundant type of oligomer seen experimentally [13], depends on the local structural rearrangement of a portion of the protein. Simulations which include two conformations (chain A and chain B) show a lower oligomerization propensity than simulations which include only one conformation (chain A). This suggests that the tetramerization mechanism is based on an induced fit model rather than a conformational selection.

Looking at the encounter complexes identified in the simulations, we can hypothesize the following mechanism for the formation of the tetramer. The monomeric unit exists in solutions in a chain A type conformation. The dipolar character of chain A drives the formation of the encounter complex which is stabilized by non-polar interactions between the aliphatic residues in the hydrophobic patch. Finally, a structural rearrangement at the protein interfaces of two of the monomers, allows bound tetramers to be obtained.

The simulations performed with a graphite layer show that HFBI proteins tend to remain close to the surface due to steric and hydrophobic contribution. The proximity of the HFBI proteins to the surface, in turn increases the probability of surface adsorption, explaining the higher affinity shown by hydrophobins to hydrophobic surfaces compared to solution association [16].

## Methods

### Interaction energies and forces

The forces were computed as finite-difference derivatives of the pairwise free energies of interaction between proteins. For each pair of proteins (labeled 1 and 2), the interaction free energy, Δ*G*, was defined as:(1)

The first four terms in Eq. 1 are electrostatic terms, the fifth and sixth terms are nonpolar terms and the last two terms describe the soft-core repulsion. A detailed description and parameterization of Eq. 1 can be found in Refs. [26,31]. In equations 1 and 2, Φ are the interaction potentials, *q *are the effective charges [32], A is the solvent accessible surface area and r are the atomic coordinates. For computational efficiency, all interaction potentials, Φ, as well as the soft-core repulsion, *E_sc_*, were mapped onto grids.

In computing the interaction between a protein and the graphite surface, some of the electrostatic terms were omitted. In particular, interaction free energy, Δ*G *becomes(2)

where 1 and 2 correspond to the surface and the protein respectively.

The soft-core repulsion term has the following expression(3)

where **r***_i _*is the center of atom *i *of radius *a_i _*and **r **is a given grid point. The term  removes the singularity at |**r **- **r***_i _*| = 0 and gives a smooth function. The value of *σ *can be tuned to vary the smoothness of the function, keeping its asymptotic behaviour. *γ *is a parameter to rescale the magnitude of the function and *nexp *is the exponent to set the decay. Details of these parameters can be found in ref. [26].

In simulations with only proteins the parameters were set to *nexp *= 6, *σ *= 3.0 Å, *γ *= 64.0 kcal/mol as described in ref. [26]. For the simulations which include the surface, the smoothness of the potential was increased by rescaling the parameter *σ *to 1.5 Å. This was necessary because of the higher probability of the proteins to remain trapped between the surface and other proteins close to the surface, which increases the probability of clashes. To compensate the larger inter-penetrability of the proteins, which leads to a stronger short-range attraction, we adjusted the non-polar desolvation term. The non-polar interaction is due to the burial of the solvent accessible surface areas of the surface atoms of one protein by the other protein. This includes a factor *β *for converting the calculated buried area to hydrophobic desolvation energy [31], which was set to *β *= -0.018 kcal mol^-1 ^Å^-2 ^in protein only simulations and to *β *= -0.013 kcal mol^-1 ^Å^-2 ^for protein-graphite simulations. These values are within the range used in ref. [26,31] for protein-protein interactions. One microsecond test simulations showed that a value of *σ *of 1.5 Å with a value of *β *within this range could be used for the simulations of the protein solutions and could reproduce radial distribution functions and diffusion coefficients.

### System preparation

The crystal structure of HFBI was taken from the Protein Data Bank (ref): 2FZ6[12]. The crystallographic structure is tetrameric. The 4 conformationally non-identical chains in the asymmetric unit can be grouped into two types based on the conformation of the second *β*-hairpin (loop 60-66) [12]. In particular, one conformation is presented by chains A and C which have a C*_α _*RMSD of 0.45 Å and the second conformation is shared by chains B and D which possess an extended conformation of the second *β*-hairpin as well as some other subtle differences since the C*_α _*RMSD between B and D is 1.00 Å. Superimposing molecule A or C with B or D gives an average C*_α _*RMSD of 2.53 Å[12].

The conformation of molecule A was used as representative for chains A and C while the conformation of chain B was used to represent chains B and D. The physical parameters of chain A and chain B are listed in Table 1.

**Table 1:** Physical properties of monomeric HFBI

Chain	No. Res.	(Å^2^/*ns*)	(*rad*^2^/*ns*)	*R_g _*(Å)	*R_h _*(Å)
A	72	13.91	4.04×10^-2^	11.89	15.75
B	69	13.69	3.81×10^-2^	12.26	16.05

Physical properties of chain A and B in the crystal structure of HFBI. (PDB code 2FZ6[12]). Each monomer has 75 residues. The number of residues given in the table corresponds to the number of residues resolved in the crystal structure of the tetramer. Translational () self-diffusion, rotational () diffusion, radius of gyration (*R_g_*) and hydrodynamic radius (*R_h_*) were computed using HYDROPRO [42] The superscript 0 on the translational and rotational diffusion coefficients indicates that these are infinite dilution values, computed considering the crystal structure of the monomer (chain A or chain B) in solution using the rigid-particle formalism [42,43].

Polar hydrogens were added to the structures according to the specified pH and ionic strength using H++ [33]. All simulations were performed at pH = 5 and IS = 50 mM. In these conditions, the net charge is zero for both chain A and chain B.

Partial charges and radii were assigned to all the atoms from the OPLS force field [34]. Electrostatic potential grids Φ were computed by solving the linearized Poisson-Boltzmann equation using UHBD [35]. The grid size was set to 100 Å with a grid spacing of 1.0 Å. In the protein-protein simulations, electrostatic and non-polar desolvation grids of HFBI were set to 80 Å, grid spacing 1.0 Å. The soft-core repulsion grid size was set to 60 Å and grid spacing 1.0 Å.

A three layer Highly Ordered Pyrolytic Graphite (HOPG) was generated using a python script [36]. The size of the surface was set to 200 × 200 Å^2^. Non-polar desolvation, electrostatic desolvation and soft-core repulsion grids were set to 200 × 200 × 60 Å^3 ^with a grid spacing of 0.5 Å.

### Brownian dynamics simulations

The positions and orientations of the particles were propagated using the Ermak-McCammon [37] algorithm.

BD simulations were carried out using 16 or 512 proteins that were initially randomly positioned (avoiding overlaps) in a rectangular box with periodic boundary conditions. The dimensions of the box were varied according to the concentration of the protein solution. In the case of protein-surface simulations, the surface was placed at the bottom of the simulation box and we considered periodic boundaries for the sides of the box and reflective boundaries for the top of the simulation box.

Each system was subjected to 10 *μ*s of simulation at 300 K. Equilibration was assessed by monitoring the convergence of the radial distribution function and the stabilization of the energies. In all cases, 1 *μ*s was sufficient to obtain an equilibrated system according to these criteria and the remaining 9 *μ*s were used for the analysis. The integration timestep was 0.5 ps. The positions and orientations of the proteins were recorded along with energy values every 0.5 ns.

BD simulations were performed with SDAMM [26], a parallelized program based on the SDA software [38] capable of handling many proteins (10^3 ^-10^4 ^) treated as rigid bodies in atomic detail.

For further details, see [26].

### Oligomer analysis and clustering: computational details

An average fraction of each oligomeric species was computed by recording the occurrence of the oligomeric states at each step of the simulation and then averaging over the total number of steps. An oligomer is defined as a group of two or more proteins which are in contact with each other. A contact is defined following the "atomic contact criterion" for the definition of encounter complexes described in Ref [39]. Namely, an encounter complex is formed when at least *N^ind ^*independent contacts between two proteins occur. A contact is established when the centers of two atoms (one from each protein) are closer than a given cutoff, *dc*. The independence of the contacts is ensured by considering only atoms within the protein that are further from each other than a distance, *d_min_*. Following Ref. [39], we set *N^ind ^*= 2, *d_c_*= 4.5 Å and *d_min _*= 6.0 Å. Cluster analysis was carried out to find the most favorable orientations in each oligomeric species. We first superpose all oligomers by least square fitting on one reference chain (e.g. for tetramers, chain A was used as the reference chain). A distance matrix was obtained by computing the root mean square (rms) distance between all pairs of the oligomers (e.g. all tetramers). The rmsd was computed for all atoms of the complete oligomeric structure. The most similar oligomers were grouped together using the gromos clustering algorithm from the GROMACS software [40] with a cutoff of 15.0 Å. In addition to describing the tendency of each atom to be involved in a contact with another protein, the number of times an atom *i *was found within *d_c _*= 4.5 Å (protein-protein) or *d_c _*= 4.0 Å (protein-surface) of an atom of another protein was counted . A relative atomic contact probability was then obtained as .

Particularly relevant residues involved in a protein-protein or protein-surface interaction were identified by setting a threshold in the atomic contact probability. In particular, a residue was considered relevant if any of its atoms has a probability greater than the third quartile of the atomic contact probability distribution.

### Protein distributions

The distribution of proteins with respect to the surface was measured by computing a surface-protein distribution function averaged over the x-y dimensions and normalized by the bulk density.

## Abbreviations

HOPG: Highly Ordered Pyrolytic Graphite; HFBI: Hydrophobin I; BD: Brownian Dynamics; IS: Ionic strength; Φ*_el_*(**r**): electrostatic potential; Φ*_ed_*(**r**): electrostatic desolvation field; Φ*_np _*(**r**): non-polar desolvation field; *E_sc _*(**r**): soft-core repulsion;

## Authors' contributions

PM performed the computational work. All authors contributed to the design of the study. Both authors read, revised and approved the final manuscript.
